# Intra-Axial Cerebral Schwannoma in a Child: A Case Report

**DOI:** 10.3390/neurolint18080145

**Published:** 2026-07-29

**Authors:** Adam M. Abdallah, Atef F. Hulliel, Bayan Maraqa, Mouness Obeidat

**Affiliations:** 1 Department of Surgery, King Hussein Cancer Center, Amman 11941, Jordan; aa.18095@khcc.jo; 2 Faculty of Medicine, Jordan University of Science and Technology, Irbid 22110, Jordan; 3 Department of Pathology and Laboratory Medicine, King Hussein Cancer Center, Amman 11941, Jordan; bm.11034@khcc.jo

**Keywords:** schwannoma, intra-axial, brain, case report, cerebral

## Abstract

Background: Primary intra-axial cerebral schwannomas are exceptionally rare benign tumors that arise within the brain parenchyma without any association with cranial nerves. Their nonspecific clinical and radiological features frequently mimic high-grade gliomas, making preoperative diagnosis challenging. Case Presentation: We report the case of a 17-year-old male who presented with recent-onset right-sided visual disturbance and bilateral early papilledema. Magnetic resonance imaging demonstrated a lobulated, contrast-enhancing left occipitoparietal intra-axial mass with restricted diffusion, hyperperfusion, extensive vasogenic edema, and adjacent calvarial remodeling, raising suspicion for glioblastoma, gliosarcoma, or pleomorphic xanthoastrocytoma. The patient underwent gross-total resection through a left occipital craniotomy. Histopathological examination revealed a well-circumscribed cellular spindle-cell neoplasm with alternating hypercellular and hypocellular areas, perivascular hyalinization, and an absence of mitotic activity or necrosis. Immunohistochemistry demonstrated diffuse positivity for S100 and SOX10, negative Olig2 staining, retained INI-1 expression, and a low Ki-67 proliferation index of approximately 5%, establishing the diagnosis of a WHO grade 1 cellular schwannoma. Conclusions: Intra-axial cerebral schwannoma should be considered in the differential diagnosis of enhancing supratentorial brain lesions in children and adolescents, particularly when imaging suggests a high-grade glioma. Definitive diagnosis relies on histopathological and immunohistochemical evaluation. Gross-total resection is associated with excellent outcomes and durable disease control, although continued radiological surveillance remains advisable given the rarity of the condition.

## 1. Introduction

Central nervous system (CNS) tumors are a significant cause of neurological morbidity and mortality across all age groups and represent one of the most commonly diagnosed categories of solid tumors in children, adolescents, and young adults [1]. Among the histological subtypes encountered, peripheral-nerve-sheath tumours represent a distinct and clinically meaningful subset, of which the schwannoma is the prototype [2]. Schwannomas are benign neoplasms that arise from the myelinating Schwann cells of the peripheral nervous system and are composed of differentiated, well-organised spindle cells. Within the cranial cavity, the overwhelming majority arise from cranial nerves, most characteristically the vestibular division of the eighth cranial nerve, where they form the prototypical vestibular schwannoma that dominates clinical experience of intracranial nerve-sheath disease [2,3].

Intracranial schwannomas account for approximately 5–8% of all primary intracranial tumours, and the vestibular schwannoma is the most common nonmalignant nerve-sheath tumour of the CNS, with a reported population-based incidence on the order of one to two cases per 100,000 person-years and a rising rate of detection attributable to increasingly sensitive magnetic resonance imaging (MRI) [4]. Irrespective of anatomical site, schwannomas are classified as World Health Organization (WHO) grade 1 tumours and only exceptionally undergo malignant transformation [2].

Compared with the typical nerve-associated schwannomas, those that arise entirely within the brain parenchyma without any identifiable attachment to cranial or spinal nerves, referred to as intracerebral, intraparenchymal, or intra-axial schwannomas, are extraordinarily uncommon. They are estimated to represent fewer than 1% of all intracranial schwannomas, with fewer than a hundred cases reported in the literature so far [5]. Early clinicopathological series describe these lesions as usually well-circumscribed, often cystic, predominantly located in the supratentorial compartment, and generally slow-growing with an indolent clinical course.

Because Schwann cells are not normally found within the mature brain parenchyma, the origin of intracerebral schwannomas remains uncertain, and several developmental and non-developmental hypotheses have been proposed to explain their rare occurrence [6]. Clinically and radiologically, these tumors are non-specific in appearance. They typically show strong contrast enhancement and are often surrounded by vasogenic edema, making preoperative diagnosis extremely challenging. As a result, they are most commonly mistaken for more aggressive intra-axial tumors such as gliomas or other glial neoplasms [7]. Occurrence in the occipital lobe with associated visual symptoms is particularly uncommon and has only been rarely reported in the literature [8].

In this context, we present a case of a primary intra-axial cellular schwannoma located in the left occipitoparietal region of a 17-year-old male who presented with right-sided visual disturbance and early bilateral papilledema. Preoperative imaging strongly favored a diagnosis of high-grade glioma. The patient underwent gross-total resection and achieved full visual recovery, remaining recurrence-free at ten months of follow-up.

Given the rarity of this entity in the pediatric and adolescent population, its tendency to mimic malignant glial tumors on imaging, and the crucial role of immunohistochemistry in reaching the correct diagnosis, we describe the clinical, radiological, and pathological features of this case and review the relevant literature.

## 2. Case Presentation

A 17-year-and-6-month-old male patient presented with recent-onset right-sided visual disturbance, and subsequent neuroimaging showed a newly discovered left occipitoparietal brain lesion on magnetic resonance imaging (MRI). He was born via normal vaginal delivery, with no history of neonatal intensive care unit (NICU) admission. His vaccinations were up to date, and developmental milestones were appropriate for age.

On ophthalmologic examination, corrected visual acuity was 0.8 in both eyes. Color vision was intact (9/9 OD, 9/9 OS), extraocular movements were full, and no relative afferent pupillary defect (RAPD) was detected. Fundoscopic evaluation revealed bilateral temporal blurring of the optic disc margins, while the maculae appeared healthy. Optical coherence tomography (OCT) of the optic nerve head revealed early papilledema bilaterally. Visual field testing was normal.

**Figure 1:**
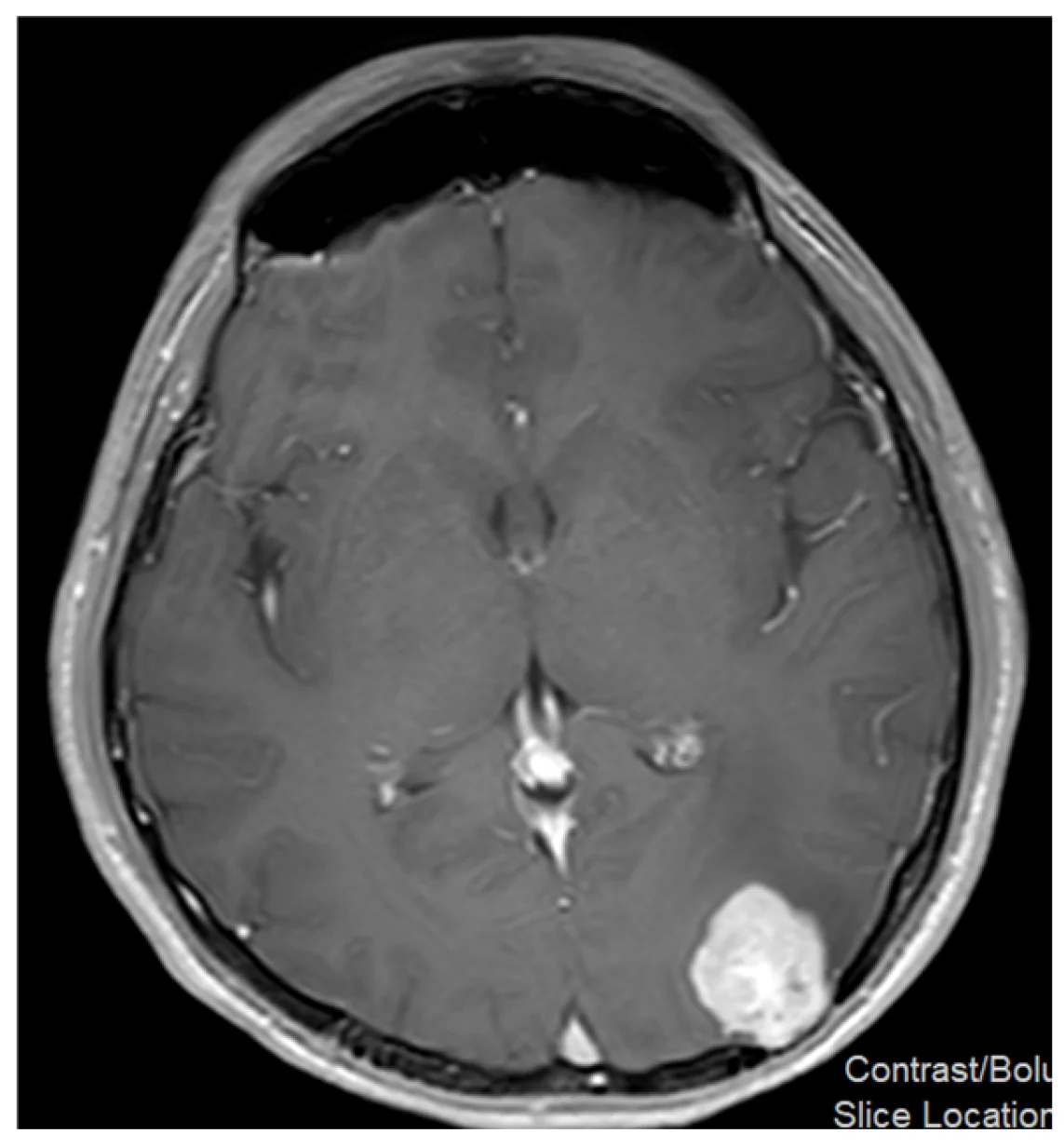
Preoperative brain MRI showing a lobulated, peripherally located, contrast-enhancing intra-axial mass in the left occipitoparietal region.

Preoperative brain MRI demonstrated a lobulated, peripherally located contrast-enhancing mass lesion in the left occipitoparietal region (Figure 1).

The lesion showed restricted diffusion and hyperperfusion, with extensive surrounding vasogenic edema, resulting in significant remodeling and thinning of the adjacent bone without definite subperiosteal extension. There was no evidence of cerebrospinal fluid (CSF) dissemination or spinal involvement. Based on radiologic characteristics, the differential diagnosis included glioblastoma, gliosarcoma, and pleomorphic xanthoastrocytoma.

**Figure 2:**
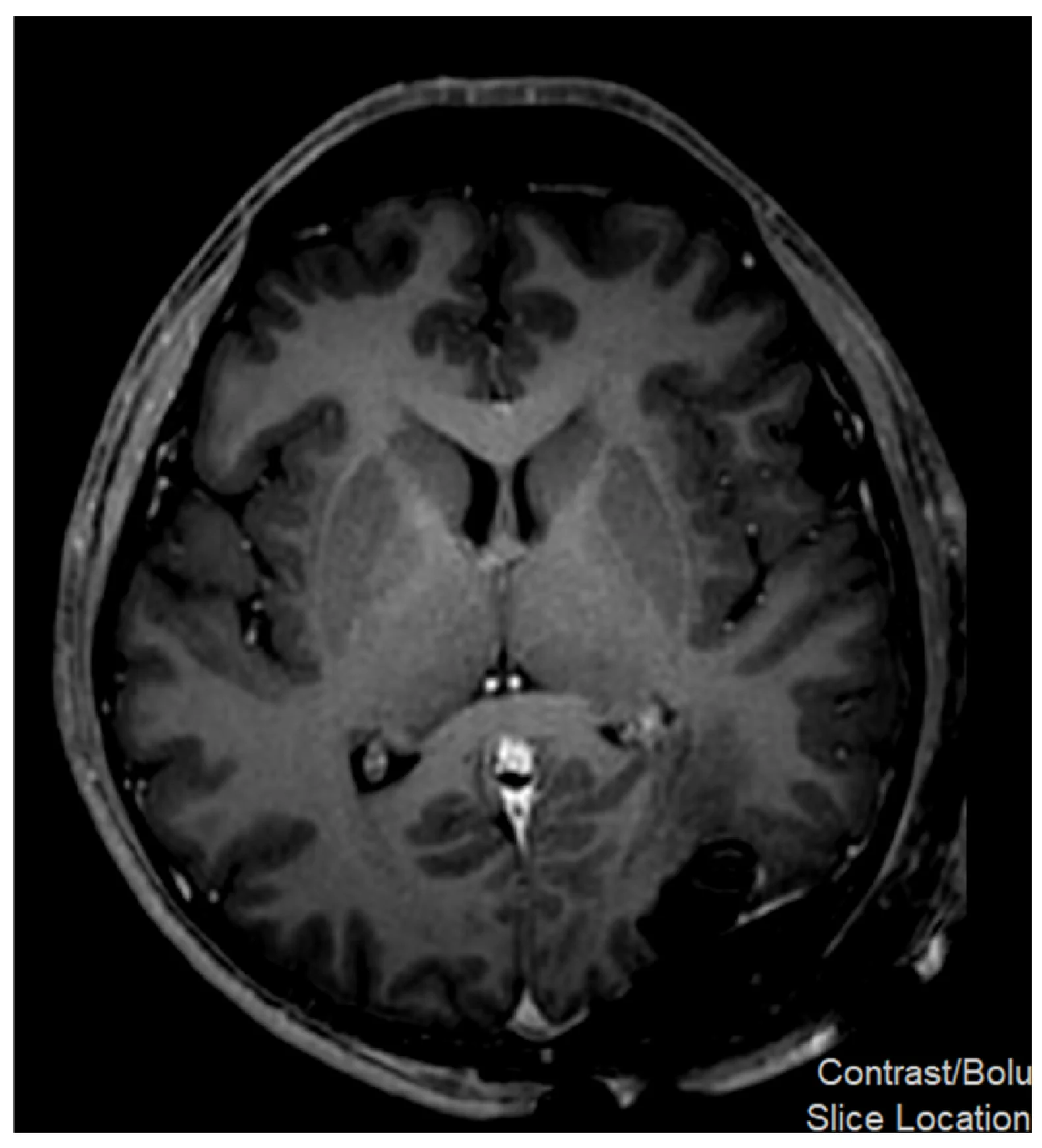
Postoperative brain MRI demonstrating complete excision of the left occipitoparietal lesion with no residual contrast enhancement.

The patient underwent a left occipital craniotomy with gross total resection of the lesion. Intraoperatively, the tumor was located within the brain parenchyma and was accessed through the cortical surface. No dural attachment or extra-axial component was identified. A clear dissection plane between the tumor and the surrounding brain tissue allowed complete excision. Postoperative MRI confirmed complete excision with no residual contrast enhancement and no newly identified lesions (Figure 2). The postoperative course was uneventful, and the patient remained neurologically intact.

**Figure 3:**
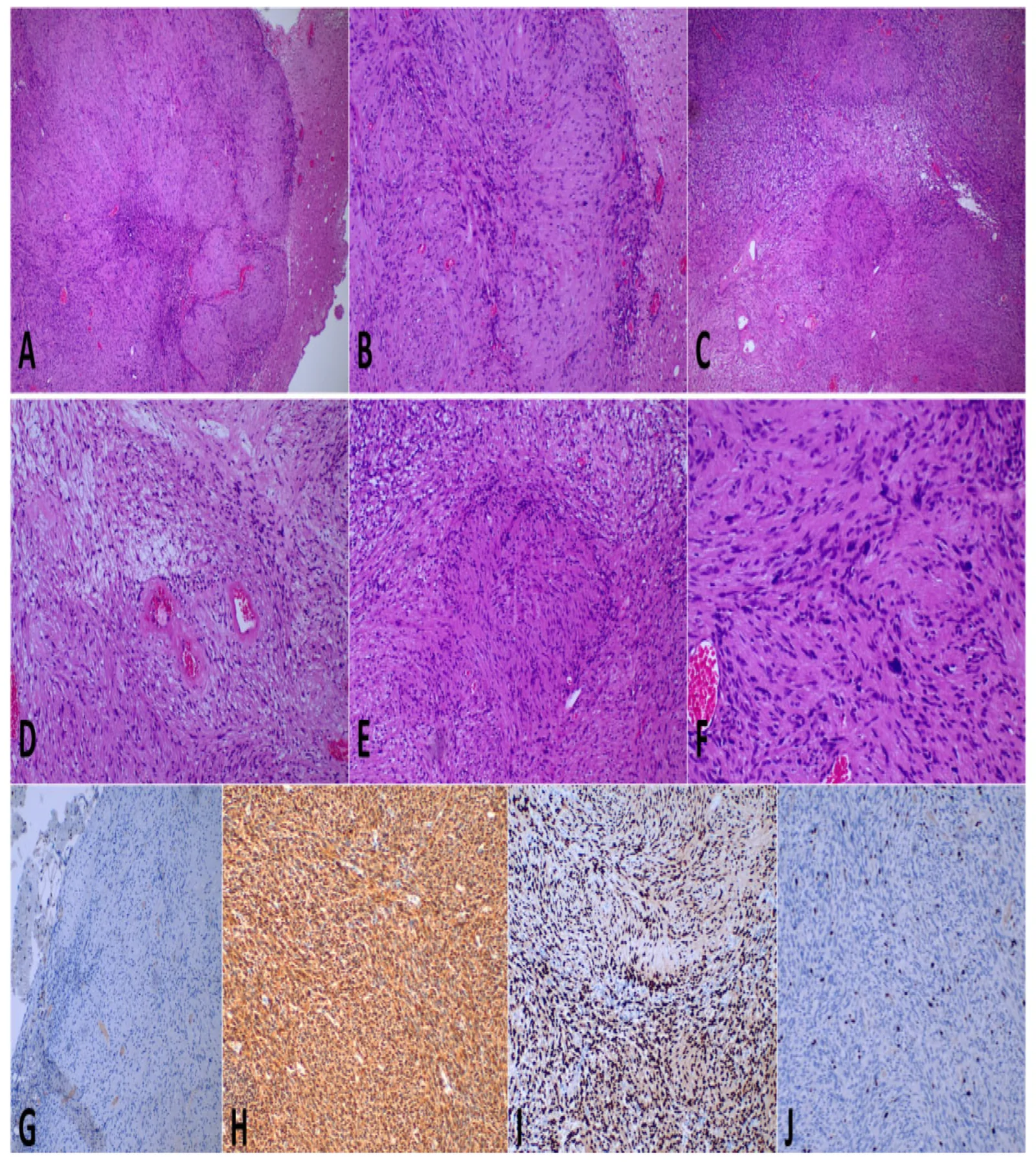
(**A**–**J**): Histopathologic examination of the tumor showing a well-circumscribed intraparenchymal neoplasm with sharp demarcation from adjacent brain tissue ((**A**,**B**,**E**,**H**), 4× and 10×), alternating hypercellular and hypocellular areas ((**C**,**E**,**H**), 4×), representative high-power fields with perivascular hyalinization, Verocay bodies, and degenerative nuclear atypia ((**D**–**F**,**H**), 40×), negative Olig2 immunostaining in tumor nuclei with positivity in surrounding brain tissue ((**G**), 40×), diffuse S100 and SOX10 positivity ((**H**,**I**), 40×), and a low Ki-67 labeling index ((**J**), 40×).

Histopathological examination of the resected (3 × 2 × 2.5 cm) mass revealed a well-circumscribed intraparenchymal neoplasm with a sharp demarcation from the adjacent brain parenchyma (Figure 3A,B). Microscopically, the tumor was predominantly cellular, composed of intersecting fascicles of spindle cells with alternating hypercellular and hypocellular areas (Figure 3C). Prominent degenerative changes, including central and perivascular hyalinization and scattered foamy histiocytes, were observed (Figure 3D–F). Tumor cells exhibited bland cytologic features with mild degenerative nuclear atypia, without significant pleomorphism, mitotic activity, or necrosis.

Immunohistochemical analysis demonstrated diffuse and strong positivity for S100 protein (Figure 3H) and SOX10 (Figure 3I), confirming Schwann-cell differentiation. Olig2 immunostaining was negative in tumor cells and positive in the surrounding brain tissue (Figure 3G), excluding a glial origin. INI-1 expression was retained, and the Ki-67 proliferation index was low, labeling approximately 5% of tumor cell nuclei (Figure 3J). Overall, the histomorphologic and immunophenotypic findings were diagnostic of a cellular schwannoma, WHO Grade 1.

Postoperatively, the patient remained neurologically intact and was discharged in good clinical condition. Serial follow-up demonstrated favorable clinical and radiological evolution. Ten months after surgery, the patient remained asymptomatic with no new neurological deficits. Visual function had improved, and ophthalmologic examination showed complete resolution of papilledema with normalization of the optic discs and preserved visual acuity. The patient remained off medications and returned to normal daily activities. Management was discussed in a multidisciplinary pediatric neuro-oncology meeting, and the plan consisted of clinical observation with periodic MRI surveillance. Genetic testing was performed, which identified a heterozygous pathogenic MUTYH variant (c.463-2A>G) on a multigene cancer panel; however, the result was considered clinically non-conclusive regarding tumor predisposition. The patient continues to undergo radiological follow-up every six months.

## 3. Discussion

We describe a 17-year-old adolescent who presented with recent-onset right-sided visual disturbance and was found to have a lobulated, peripherally located, contrast-enhancing intra-axial mass in the left occipitoparietal region, with restricted diffusion, hyperperfusion, and extensive vasogenic oedema and remodelling of the adjacent calvarium. Ophthalmological assessment, including optical coherence tomography, demonstrated bilateral early papilledema. On these grounds, the preoperative differential diagnosis comprised glioblastoma, gliosarcoma, and pleomorphic xanthoastrocytoma.

Although restricted diffusion and hyperperfusion are commonly associated with high-grade gliomas, these radiological features are not entirely specific for malignant tumors and may also occur in benign tumors. In particular, cellular schwannomas may demonstrate diffusion restriction because of their densely packed spindle-cell architecture, resulting in reduced extracellular space and limited water mobility [9]. Similarly, increased perfusion may reflect the presence of prominent intratumoral vasculature and variable vascular proliferation within schwannomas rather than aggressive biological behavior. Therefore, in rare intracerebral schwannomas, advanced MRI characteristics may mimic those of high-grade glioma and contribute to diagnostic uncertainty [9,10]. Definitive diagnosis relies on histopathological evaluation and immunohistochemical confirmation of Schwann-cell differentiation.

The demographic and anatomical features of our patient are in line with what has been described in the literature on this rare entity. Published reports and reviews consistently show that intraparenchymal schwannomas tend to affect children and young adults more frequently and are most often located in the supratentorial compartment [11]. For instance, a previously reported 16-year-old patient with a mixed cystic–solid frontoparietal intracerebral schwannoma closely parallels our case in age [11], and similar pediatric cases presenting with seizures and successfully treated by gross-total resection have also been documented [12]. Even more unusual presentations, such as intraventricular schwannomas in children, further highlight the variability in their intracranial distribution in younger patients [13].

In contrast, the occipital location and the predominantly visual symptoms observed in our patient are distinctly uncommon. Such a presentation has been reported only rarely, and broader reviews of intracerebral schwannomas continue to show that seizures and headaches remain the most frequent clinical manifestations [14].

The pathogenesis of intracerebral schwannoma remains largely speculative, largely because Schwann cells are not normally present within the brain parenchyma. As outlined in previous reviews, two main explanatory frameworks have been proposed: developmental theories, which suggest aberrant migration or misplacement of neural crest-derived cells during embryogenesis, and non-developmental theories, which propose an origin from Schwann cells associated with perivascular nerve plexuses of subarachnoid or intraparenchymal vessels [11,15]. In our case, the purely intraparenchymal location, the well-circumscribed nature of the lesion, and the absence of any dural or cranial nerve attachment at surgery collectively favor a primary parenchymal origin rather than secondary extension. The patient’s adolescent age further provides indirect support for a developmental mechanism.

Immunohistochemistry played a pivotal role in establishing the correct diagnosis and excluding radiological mimics. The tumour showed strong, diffuse co-expression of S100 protein and SOX10, confirming Schwann-cell differentiation and aligning with the well-established utility of these markers in identifying Schwannian and neural crest lineages [16]. Importantly, Olig2 was completely negative in the tumour cells while appropriately highlighting the entrapped surrounding glial tissue; given the near-universal expression of Olig2 in diffuse gliomas, its absence effectively ruled out a glial neoplasm and contradicted the preoperative impression of high-grade glioma. Retained INI-1 expression together with a low Ki-67 proliferation index further supported a benign nerve sheath tumour.

Despite its benign nature, the lesion exhibited hypercellularity, fascicular architecture, and areas of nuclear atypia, features that necessitated careful exclusion of malignant peripheral nerve sheath tumour (MPNST), a known diagnostic pitfall in cellular schwannoma. Prior clinicopathological studies have highlighted several features favoring cellular schwannoma over MPNST, including foamy macrophage infiltration, hyalinized thick-walled vessels, preserved SOX10 expression, and, most importantly, a low Ki-67 index, typically well below the ~20% threshold associated with malignancy, with essentially no reported metastatic behavior in true cellular schwannomas [17]. In our case, the combination of retained SOX10, preserved INI-1, a Ki-67 index of approximately 5%, and the absence of mitotic activity or necrosis strongly supports a benign diagnosis. This distinction is clinically critical, as malignant intracranial nerve sheath tumours, although exceedingly rare, may occur sporadically or in the setting of tumor predisposition syndromes and are associated with a significantly worse prognosis [18].

Genetic testing identified a heterozygous pathogenic MUTYH variant (c.463-2A>G) on a multigene cancer panel. This finding was considered incidental and not clinically explanatory in relation to the present tumour. Biallelic MUTYH mutations are known to cause MUTYH-associated polyposis, an autosomal recessive colorectal cancer predisposition syndrome, whereas monoallelic carriers may have only a modest and still debated increase in colorectal cancer risk, with no established association with schwannomas or central nervous system tumours [19]. The solitary nature of the lesion, absence of additional schwannomas or cutaneous stigmata, and lack of relevant family history further argue against an underlying NF2 or schwannomatosis spectrum disorder, although more extensive molecular profiling was not performed.

From a therapeutic standpoint, this case reinforces the principle that complete surgical excision is curative for these lesions and is associated with excellent neurological recovery. In our patient, a well-defined surgical plane allowed complete excision, and the resolution of papilledema with recovery of visual function indicates that the deficit was due to reversible mass effect rather than permanent optic pathway injury. Given the rarity of this entity and the limited long-term outcome data, periodic MRI surveillance, here performed at six-month intervals, remains a reasonable precaution.

The strengths of this report include a thorough multimodal diagnostic evaluation, incorporating advanced MRI sequences, formal neuro-ophthalmological assessment with optical coherence tomography, and an extensive immunohistochemical panel (S100, SOX10, Olig2, INI-1, and Ki-67), alongside germline genetic testing and multidisciplinary decision-making. Objective documentation of both radiological and functional recovery further strengthens the clinical value of this case.

Several limitations should also be acknowledged. As a single case, the findings cannot be generalized or used to infer causality. The relatively short follow-up period of ten months, although reassuring, is insufficient to fully exclude late recurrence in an indolent tumor. Finally, while intraoperative findings strongly support a primary parenchymal origin, with no evidence of dural attachment, cranial nerve involvement, or an identifiable extra-axial component, an origin from an occult microscopic intraparenchymal nerve twig cannot be entirely excluded.

In conclusion, primary intra-axial cellular schwannoma should be considered in the differential diagnosis of an enhancing supratentorial mass in children and adolescents, particularly when imaging suggests a high-grade glioma. Accurate diagnosis relies heavily on immunohistochemistry, with a characteristic profile of diffuse S100 and SOX10 positivity, Olig2 negativity, retained INI-1 expression, and a low proliferative index, which together effectively exclude both diffuse gliomas and malignant nerve sheath tumours. Gross-total resection is curative in most cases and may lead to complete neurological recovery, as demonstrated in this patient, although continued radiological follow-up remains advisable given the rarity of the entity.

## 4. Conclusions

Primary intra-axial cellular schwannoma is an exceptionally uncommon intracranial neoplasm that can closely mimic high-grade gliomas both clinically and radiologically. This case highlights the diagnostic challenges posed by these lesions, particularly in children and adolescents presenting with enhancing supratentorial masses and significant surrounding edema. Accurate diagnosis depends on careful histopathological assessment supported by immunohistochemical findings, including diffuse S100 and SOX10 positivity, Olig2 negativity, retained INI-1 expression, and a low proliferative index. Gross-total surgical resection remains the treatment of choice and is associated with excellent neurological recovery and a low risk of recurrence. Awareness of this rare entity may help avoid diagnostic misclassification and guide appropriate management. Despite its benign behavior, continued radiological follow-up is prudent because long-term outcome data remain limited.

## 5. Patents

All subjects participated voluntarily, and all procedures were conducted in accordance with ethical standards.

## Data Availability

The datasets used and/or analysed during the current study are presented in tables and text.
